# Feasibility of PROMIS using computerized adaptive testing during inpatient rehabilitation

**DOI:** 10.1186/s41687-023-00567-x

**Published:** 2023-05-10

**Authors:** Riyad Bin Rafiq, Susan Yount, Sara Jerousek, Elliot J. Roth, David Cella, Mark V. Albert, Allen W. Heinemann

**Affiliations:** 1 Department of Computer Science and Engineering, University of North Texas, Denton, TX 76201 USA; 2 Department of Medical Social Sciences, Feinberg School of Medicine, Northwestern University, Evanston, USA; 3 Ann & Robert H. Lurie Children’s Hospital, Chicago, USA; 4 Department of Physical Medicine and Rehabilitation, Feinberg School of Medicine, Northwestern University, Evanston, USA; 5 Department of Biomedical Engineering, University of North Texas, Denton, USA; 6 Center for Rehabilitation Outcomes Research, Shirley Ryan AbilityLab, Chicago, USA

**Keywords:** Patient-reported outcomes (PROs), Inpatient rehabilitation, Computerized adaptive testing (CAT), Patient-reported outcomes measurement information system (PROMIS)

## Abstract

**Background:**

There has been an increased significance on patient-reported outcomes in clinical settings. We aimed to evaluate the feasibility of administering patient-reported outcome measures by computerized adaptive testing (CAT) using a tablet computer with rehabilitation inpatients, assess workload demands on staff, and estimate the extent to which rehabilitation inpatients have elevated T-scores on six Patient Reported Outcomes Measurement Information System® (PROMIS®) measures.

**Methods:**

Patients (N = 108) with stroke, spinal cord injury, traumatic brain injury, and other neurological disorders participated in this study. PROMIS computerized adaptive tests (CAT) were administered via a web-based platform. Summary scores were calculated for six measures: Pain Interference, Sleep Disruption, Anxiety, Depression, Illness Impact Positive, and Illness Impact Negative. We calculated the percent of patients with T-scores equivalent to 2 standard deviations or greater above the mean.

**Results:**

During the first phase, we collected data from 19 of 49 patients; of the remainder, 61% were not available or had cognitive or expressive language impairments. In the second phase of the study, 40 of 59 patients participated to complete the assessment. The mean PROMIS T-scores were in the low 50 s, indicating an average symptom level, but 19–31% of patients had elevated T-scores where the patients needed clinical action.

**Conclusions:**

The study demonstrated that PROMIS assessment using a CAT administration during an inpatient rehabilitation setting is feasible with the presence of a research staff member to complete PROMIS assessment.

## Introduction

Patient-reported outcomes (PROs) have been embraced increasingly as relevant endpoints of clinical trials and clinical research as well as routine clinical practice [1, 2]. In the United States, the current focus on patient-centered research and care has reinforced the value of PROs and the inclusion of the patient’s voice in research [3, 4]. There is a growing body of evidence that PROs contribute to improved quality of care and patient-provider communication [5, 6], aid in the management of chronic conditions [7], screen for specific health disorders [8, 9], highlight symptoms that providers systematically miss or underappreciated [10, 11], and increase patient satisfaction with care [12]. PROs have also been predictive of distal outcomes such as disease status, mortality, morbidity, and function in a range of conditions and diseases that are objective measures of disease [13–15]. There is also growing interest in the use of PROs in clinical care for measuring facility performance [16], particularly for surgical care, as they provide complementary information to typically monitored clinical outcomes such as morbidity and mortality [17].

Several other factors have contributed to the rapid increase in the adoption and implementation of PROs in clinical settings, including the application of item response theory (IRT) measurement to the development of PROs [18–20], and progress within the technological infrastructure, allowing for wider use of information technology for PRO administration, scoring, display of results, and interpretation via tablet computers [21–23], smartphone apps [24–26], and electronic medical records [27, 28]. In addition, there is an increased demand by payers, accreditors, professional organizations, and clinicians to measure and improve PRO-measured outcomes at the patient, clinic, and healthcare system levels [29].

Most of the literature reporting on the use of PROs in rehabilitation-focused clinical settings has focused on outpatient ambulatory clinics or the transition from inpatient to outpatient settings [30–32]. In other clinical settings, such as oncology, the feasibility of patients reporting during outpatient clinic visits via waiting room tablet computers has been established, with mean compliance rates ranging from 75 to 85%, high patient satisfaction, and good usability of systems even among those who are not familiar with the internet, are elderly, or frail [33, 34].

PROs have also been used in inpatient settings, although the literature is considerably sparse in this context [35]. The assessment of hospitalized patients may pose additional challenges and require additional resources, as these patients are likely to require assistance in completing measures [36]. Depending on the clinical population being assessed, there may be cognitive, communication, and physical challenges ranging from intravenous lines in arms to functional limitations that limit PRO data collection [21, 37]. Patients in acute care settings typically have short stays, requiring consideration of the timing and frequency of PRO administration [1, 38]. The hospital environment may influence their responses [39] or render the content of the PRO items and their response categories irrelevant. Conversely, patients hospitalized in rehabilitation settings have longer stays and structured schedules, allowing easier integration of PRO assessments into their daily routine.

The importance of PROs to rehabilitation research and clinical practice has been noted, as has the value of obtaining outcomes-related data from patients themselves [40–42]. Standardized PRO measures can be used to assist in the clinical care of patients for several purposes: to determine an individual’s strengths and weaknesses; to facilitate effective interdisciplinary communication; to determine readiness to move to the next level of rehabilitation or discharge from inpatient care; and following discharge, to track functional independence, participation, health status, and health-related quality of life [43, 44]. Beyond clinical care, PROs have a role in rehabilitation comparative effectiveness research, clinical trials [45], and in assessing provider performance.

There are important considerations in the choice and implementation of PROs in rehabilitation assessment [46, 47]. These considerations include content and response formats of the questions, such as time references that might not be relevant for individuals with highly variable symptoms. Rehabilitation populations often have unique barriers to completing PROs that need to be addressed. For example, stroke patients may have cognitive, communication, or other functional deficits, limiting them from completing PROs or answering items in a reliable manner [48]. Assessment by a proxy may substitute for patient self-assessment [49, 50], although differences between patient and proxy reports suggest proxy reports should be considered complementary and not a substitute [51]. Older rehabilitation inpatients may have comorbid conditions, which argues for measures that capture health status across the conditions, to the extent possible, as opposed to the burden of additional measures [52, 53]. In addition, PRO assessment in rehabilitation settings faces the challenge common to most clinical settings: the absence of a widely accepted measure and lack of consensus regarding which measures to use [54, 55].

The Patient Reported Outcomes Measurement Information System (PROMIS), initiated by the National Institutes of Health (NIH) in 2004, is a collection of person-centered measures that can be used to evaluate the physical, mental, and social health of adults and children, both in the general population and individuals living with chronic conditions. It was developed and validated using state-of-the-science methods to be psychometrically sound and to transform how life domains are measured. Most of the measures are universal and are designed to be relevant across a wide range of conditions for the assessment of symptoms and functions. PROMIS measures are reported using a T-score metric in which 50 is the mean of a reference population and 10 is the standard deviation. In PROMIS measures, high T-scores represent more of what is being measured. For example, high T-scores of fatigue mean a severe level of fatigue while high T-scores of physical function represent the good condition of the body. Most PROMIS measures are based on the mean score of a sample of individuals that matched the US 2000 General Census with respect to gender, age, race/ethnicity, and education [56].

PROMIS measures offer several valuable features: they are not disease-specific, which allows for comparisons across conditions or populations; there are several administration options, including fixed-length “short forms” and dynamic assessment using computerized adaptive testing (CAT), which allows for brief, precise assessments with a reduced respondent burden; and the item banks are constructed to cover the full range of a trait, reducing or eliminating floor and ceiling effects [57–59]. CAT is an individually-tailored test with items selected based on the patient's level on the trait being measured [60, 61]. As CAT restricts questioning to a specific number of distinct items, CAT can be more responsive than traditional, fixed-length evaluation tools and reduce response burden precisely [19].

Building on the development and methodology of PROMIS, the development of several rehabilitation-relevant measurement systems has been funded. Quality of Life in Neurological Disorders (Neuro-QoL) [62] which evaluates and monitors the physical, mental and social effects experienced by adults and children living with neurological conditions was funded by the NIH. Moreover, the Spinal Cord Injury-Quality of Life (SCI-QOL) measurement system [63]; and the Traumatic Brain Injury-Quality of Life measurement system (TBI-QOL) [64] are funded by the National Institute on Disability and Rehabilitation Research.

PROMIS measures have been used in several studies involving rehabilitation populations [65] though their use in an inpatient rehabilitation setting has been reported recently [36, 66, 67]. CAT platforms enhance treatment and decision-making for patients through the collection of PROs [68]. CAT administration is useful in collecting patients’ outcomes in primary care settings these days [69, 70]. After being discharged from inpatient rehabilitation, PROs have been usually administered by telephone interviews or mailed questionnaires. But these approaches were tedious, costly, and had low response rates from the patients [31, 71, 72]. Therefore, CAT administration was conducted to collect patient-reported outcomes for post-rehabilitation patients and the study showed that it was feasible for a subset of patients [32]. So, CAT can be another choice for increasing patient involvement and minimizing costs in order to measure PROs [73, 74]. Few studies assessed the feasibility of PRO data collection using CAT in medical rehabilitation [32]. Completion rates, acceptability, time, and type of survey administration measured the feasibility study [70]. In this study, our objective was to evaluate the feasibility of administering PROMIS measures using a tablet computer with rehabilitation inpatients by examining the burden on patients as well as the clinical staff. Feasibility was assessed by completion time, completion rate, and staff assistance required. We also sought to estimate the extent to which rehabilitation inpatients have elevated scores on PROMIS Pain Interference, Sleep Disruption, Anxiety, Depression, Illness Impact Positive, and Illness Impact Negative measures. We sought to collect and report PROMIS measures shortly before routinely scheduled team conferences to enhance clinical relevance.

## Methods

### Study approach

**Fig. 1:**
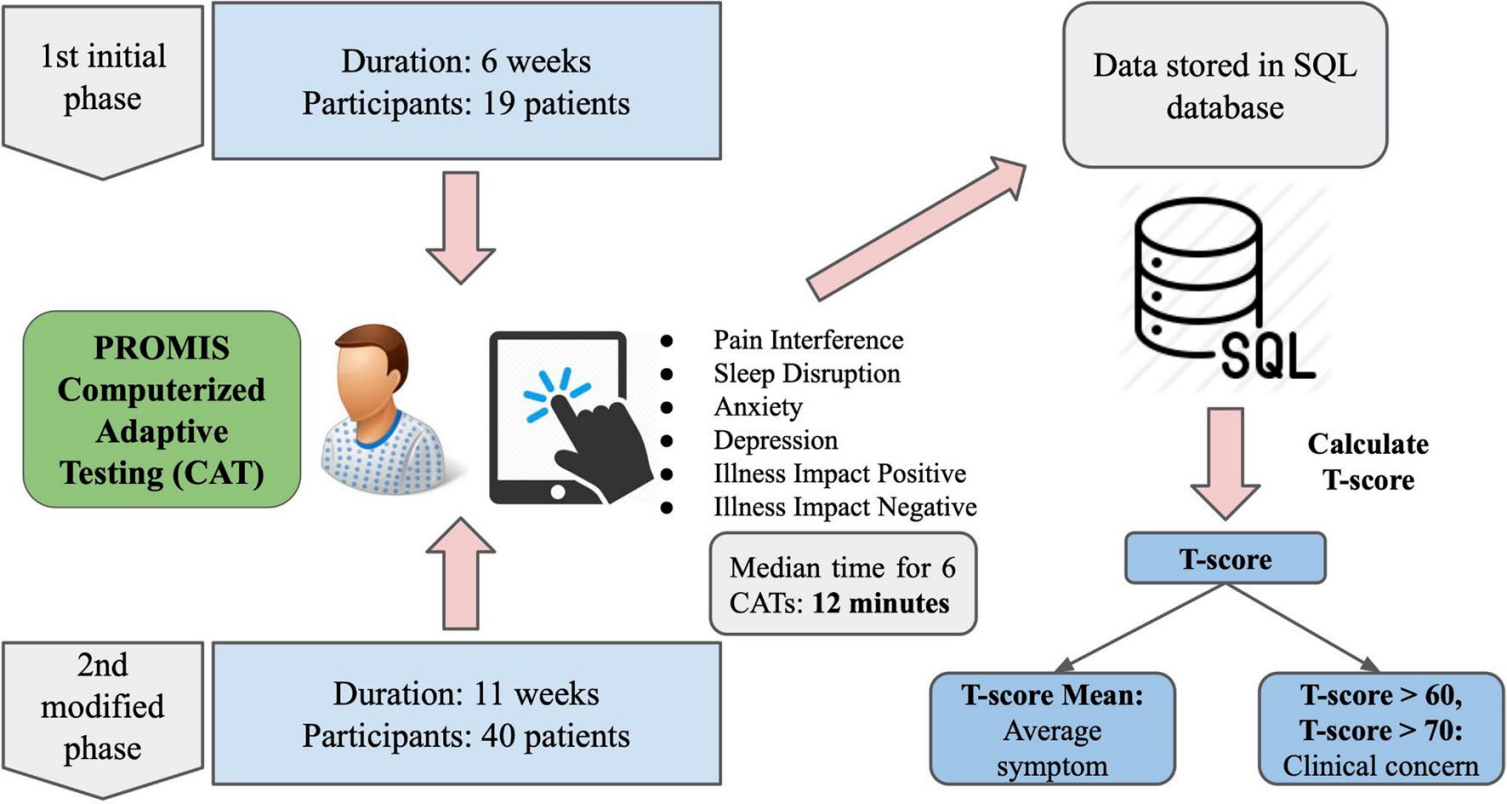
Study approach: PROMIS on pain interference, sleep disruption, anxiety, depression, illness impact positive, and illness impact negative during inpatient rehabilitation using a CAT

The Shirley Ryan AbilityLab, formerly the Rehabilitation Institute of Chicago (RIC), is an internationally recognized specialty hospital and healthcare network dedicated to the care and rehabilitation of physical and neurological disabilities. Of the 182 inpatient beds at the flagship hospital, 24 were taken for conducting the study. The floor served individuals with neurological disorders including stroke, spinal cord injury, traumatic brain injury, and other neurological disorders. Patients completed the CAT through a web-based platform using tablet computers and the data were stored in a SQL database. Finally, T-scores provide the symptom severity to the clinicians. Figure 1 depicts the study approach.

### Participants

Patients were eligible if they had a neurological disorder including stroke, spinal cord injury, traumatic brain injury, and could complete the CAT without assistance. The patients who required assistance reading items or reporting responses were coded as “not appropriate”. We excluded patients who were unavailable, refused to participate, and had other impairments including language barrier, behavioral issues, etc.

### Data collection

This study obtained input from rehabilitation physicians, nurses, and allied health therapists regarding the issues they considered most important to assess during inpatient rehabilitation. PROMIS item banks that aligned with the issues they identified were Pain Interference, Sleep Disruption, Anxiety, and Depression. Clinicians were also interested in understanding how patients perceived the negative and positive consequences of the conditions that precipitated their admissions. Thus, they recommended administering Illness Impact Positive and Illness Impact Negative. Northwestern University Department of Medical Social Sciences staff consulted with the hospital’s Information Systems department to create a local installation of the NIH Assessment Center^SM^, a web-based platform for PROMIS CAT administration and scoring, behind the hospital’s secure firewall. AbilityLab’s Information Systems staff set up a SQL database that received data from tablet computers over the hospital’s Wi-Fi network.

Our initial plan was to complete assessments the day before each patient’s weekly team conference. Nurses were charged with approaching patients and asking them to complete the assessments within 24 h of the weekly team conference as part of their routine clinical duties. They were instructed to invite patients to complete the CATs using a tablet computer and to demonstrate how to use the equipment but provided no additional assistance. The study had two phases and the sole procedural change between these phases was the substitution of a research assistant for a nursing role in instrument administration. It became evident quickly that the nursing staff was not able to administer instruments consistently and in a timely manner in addition to their other responsibilities. Even though patients are apt to have a relationship with nursing staff and be amenable to answering PRO questions, the loss of PRO data imperiled the success of the project. Thus, we decided to assign assessment responsibility to a research assistant to document the staff workload that was required.

### Implementation planning

We considered several of the questions listed in the International Society for Quality of Life Research Guide to implement PRO assessments in clinical practice [75]. We list the key decisions in Table 1.

**Table 1:** Several questions and decisions as part of the implementation planning

Question no.	Questions	Decisions
1	What are your goals for collecting PROs in your clinical practice and what resources are available?	To evaluate the feasibility and utility of PRO assessments during inpatient rehabilitation
2	Which key barriers require attention?	Lack of staff experience with PROs and uncertainty regarding the patient's ability to complete PROs
3	Which groups of patients will you assess?	Pilot test on one floor serving patients primarily with neurological disorders
4	How do you select which questionnaire to use?	Staff discussion
5	How often should patients complete questionnaires?	Weekly before team conference
6	How will the PROs be administered and scored?	CAT via the local installation of the Assessment Center
7	What tools are available to aid in score interpretation and how will scores requiring follow-up be determined?	We developed a score interpretation guide based on PROMIS documents
8	When will the results be presented?	Accessible online at any time, but shared during team conferences by the care coordinator
9	Where will the results be presented?	In a designated “huddle” room on the patient care floor
10	How will results be presented?	Via Dundas Dashboard using a large screen monitor
11	Who will receive score reports?	All team members have access to reports. Care coordinators are charged with displaying assessment results during conferences
12	What will be done to respond to issues identified through the PROs?	Results will be shared with attending physician, nursing and allied health staff members, and psychologists
13	How will the value of using PROs be evaluated?	Questionnaire via SurveyMonkey

## Results

### Implementation effectiveness

The feasibility was assessed with completion time, completion rate, and staff assistance required. Nineteen of 49 eligible patients (39%) completed an initial assessment by nurses during the first phase of the study which lasted 6 weeks. Reasons for not completing the assessments included patient unavailability (27); cognitive or communicative impairments (2), and patient sleeping (1). During the second modified phase, a dedicated staff member was appointed to assist patients with the assessments. A total of 98 assessments from 40 of the 59 admitted patients (68%) were completed. Out of the 98 completed assessments, 40 patients completed CATs on 1 occasion, 13 patients completed CATs on 2 occasions, 5 patients completed CATs on 3 occasions, 3 patients on 4 occasions, and 1 patient on 5 occasions. In this context, the patients cooperated with our staff on multiple occasions. Patients’ lengths of stay vary widely, reflecting the extent of functional improvement, goal attainment, and readiness of the family for discharge among other considerations. Thus, we had the opportunity to evaluate the feasibility of routine reassessments over varying lengths of stay. Clinicians used results from repeated assessments and changes (or stability) over time to inform clinical decision-making. Our focus was on the feasibility of repeated assessments—would patients object to or cooperate? We certainly expect within-patient repeated measures to be correlated as this is well established. The median time to complete the 6 CATs was 12 min (interquartile range = 5–46 min). This completion time in a busy inpatient rehabilitation setting proves our successful implementation as planned. Patients completed 12% of the assessments independently; the staff members read questions to facilitate 3% of the assessments and read and recorded responses for 85% of assessments. We documented PROMIS item responses regardless of staff assistance. The proportion of assessments requiring staff assistance helps inform the staffing requirements for routine PROMIS administration. Patient eligibility and recruitment status are reported in Table 2.

**Table 2:** Patient eligibility and recruitment

Recruitment status	Total patients (% patients)
*Not appropriate (n = 39)*	
Aphasia	3 (7.7)
Language barrier	3 (7.7)
Behavioral issue	1 (2.6)
Illness	1 (2.6)
Not specified/other	31 (79.4)
*Unavailable (n = 27)*	27
*Refused (n = 8)*	
Busy/family in room	1 (12.5)
Too tired	2 (25.0)
No reason given	5 (62.5)
*Patients who were approached more than once (n = 15)*	
Completed	6 (40.0)
Not completed	9 (60.0)
1 attempt	2 (13.3)
2 attempts	5 (33.3)
3 attempts	7 (46.7)
7 attempts	1 (6.7)
*Completed PROM (n = 93)*	
Independent	11 (11.8)
Minimum assistance	3 (3.2)
Maximum assistance	79 (85.0)

Minimum assistance means reading questions to facilitate the assessment. Maximum assistance means reading and recording responses to complete the assessment

### PROMIS results

During the first 6 weeks, nurses completed initial assessments. It was evident that the added burden on nurses resulted in many missed assessments. They did not have the time to make multiple attempts to complete assessments or wait for patients to wake or visitors to depart. We modified the protocol during a second phase lasting 11 weeks by designating a dedicated staff member to complete PROMIS assessments and providing assistance as needed to complete assessments. In this way, we adapted the procedure of staffing requirements for routine data collection in the rehabilitation setting and in doing so, the completion rate increased from 39 to 68%. Assistance included reading questions and recording answers as directly as possible when requested by patients. When the second phase started, we also documented the extent of assistance requested by patients. The staff member asked patients to complete the assessments and offered the minimum amount of assistance required, reading items and progressing to recording responses. Level of assistance was not a criterion since the staff time would be similar to monitoring PROMIS completion or providing assistance. We deemed the patient and staff time commitment and completion rate as satisfactory in this pilot study.

Table 3 shows that while mean T-scores were in the low 50 s, indicating an average symptom level, the score distribution was such that between 19 and 31% of patients had PROMIS T-scores with clinically actionable results (T-scores > 60), and 2% to 7% had scores more than 2 standard deviations above the mean (T-scores > 70). Most of these patients had not been identified by the clinical team as demonstrating significant clinical concerns.

**Table 3:** Descriptive statistics for PROMIS administrations

PROMIS CAT	T-score mean	Percent T > 60	Percent T > 70
Pain interference*	54.9	30.5	7.0
Psychological illness impact negative*	54.6	30.6	5.6
Psychological illness impact positive	46.5	18.5	2.4
Sleep disruption*	54.9	29.8	6.5
Fatigue*	55.3	29.8	4.8
Depression*	52.7	19.4	3.2

*In PROMIS CAT, the more T-score increases, the more severity level increases

## Discussion

The CAT usage in rehabilitation is appealing as it can reduce the burden on the respondent. In this study, the goal was to evaluate the feasibility of collecting data from patients with neurological disorders using PROMIS CAT during an inpatient rehabilitation setting. For this research, the data was collected from patients themselves by obtaining response formats of the survey questions in the assessment. Previous studies attempted PRO data collection during inpatient hospitalizations and after inpatient rehabilitation. In one study, only 7% of the eligible patients completed the CAT-administered PRO as they were using the internet or telephone after being discharged from inpatient rehabilitation [32]. In addition, completion rates for other feasibility studies during inpatient (51%) and outpatient (41%) were almost the same as ours (39% and 67% in the initial and modified phase respectively) [21, 76]. Although our rate appears to be on the low end of estimates compared to one study [77], the response rate reflects our ability to provide sufficient staffing to collect PRO data.

PROMIS item banks were developed with the general population and clinical samples. Results are reported as T-scores and have been published in a variety of peer-reviewed journals [78]. Investigators routinely use parametric statistics to compare groups and examine change over time [79]. T-scores for CAT are more reliable and have instant outcomes in a real-world scenario [80]. In addition, this scoring metric has been used in different PROMIS feasibility and comparison studies [80–82]. So, we calculated the T-score metric based on the collected data to interpret the PROMIS scores. From Table 3, we can see that all six PROMIS measures have a T-score mean (average symptom level) which is less than 60. Pain Interference has 7% of patients who had T-scores greater than 70. Again, Psychological Illness Impact Negative and Pain Interference have 30.6% and 30.5% of patients, respectively, with T-scores above 60. In this context, a higher T-score means it is important or advised to take appropriate clinical action. Conversely, for the cases of Psychological Illness Impact Positive and Depression, most of the patients had lower T-scores. In this scenario, T-score provides an overall health status for the patients who participated in the PROMIS CAT in an inpatient rehabilitation setting.

To design our feasibility study, we focused on the required key areas including acceptability, implementation, practicality, and adaptation according to the feasibility study design [83]. One aspect of the feasibility relates to patient cooperation; another relates to the staffing requirements for routine data collection. Results allow us to estimate the staffing required for routine data collection and the efforts required to complete assessments outside of therapies, meals, bowel and bladder programs, and visits by family members and friends. The added burden on nurses resulted in many missed assessments during the initial phase of the study. That’s why we modified the protocol during the second phase according to the requirements and context. Moreover, the time to complete the CAT was also acceptable in a busy inpatient setting. Therefore, results from this feasibility study provide valuable lessons that will help guide PROs collection during inpatient rehabilitation. First, clinicians identified six PROMIS item banks as relevant to patients’ concerns. While most T-scores were not in a range that required clinician action, the information could help inform patient care decisions. Following the completion of this feasibility project, clinicians decided to update the assessment protocol and omit Illness Impact Positive and Illness Impact Negative as they found information from these item banks to be less actionable.

We acknowledge some limitations in the research. Study limitations include the evaluation of PROMIS feasibility at only one inpatient rehabilitation hospital. As patients were engaged from only one rehabilitation hospital, results cannot be generalized confidently to other rehabilitation hospitals and units. However, sometimes patients couldn’t participate to complete the CAT due to their family in the room or they were sleeping. Also, the staff of the rehabilitation hospital had limited time, making it difficult to make multiple attempts to complete the assessment. In our inpatient study, it was a required step to assign a research assistant to assist patients in completing the assessments [36]. We do not believe that a research assistant would introduce markedly different bias than would a nurse—both kinds of staff members arrived with a tablet computer and asked patients to complete the PROs as independently as possible. We note that the level of assistance may create a bias. However, for the purposes of this study, the need for a nonclinical staff member to collect the data is an important finding. In our study, we couldn’t provide the observed standard deviations for each T-score. Despite these lackings, the results demonstrate how CAT assessment provides overall health status during busy rehabilitation settings within a short time. Future studies should develop a multilingual CAT platform and assign more staff members for collecting inpatient data to increase the response rate. Future research should also focus on compliance monitoring that measures patient and staff time to complete assessments in the field of the CAT platform during inpatient rehabilitation.

## Conclusions

The collected data supports that Patient-reported outcome measure (PROM) assessments using CAT are feasible during an inpatient rehabilitation setting with the presence of a research staff member to complete PROMIS assessment. We noted that the small sample limits the generalizability of the study findings. The primary focus of the study was to evaluate the feasibility of PRO data collection and estimate the resources that are required. For these purposes, the sample was sufficient. There is enough variation in patient-level outcomes to support their consideration as patient-reported outcome-based performance measures (PROM-PMs). Further work is needed to identify the frequency of patient and institutional barriers that affect the feasibility of routine assessment across patient populations during inpatient settings and the utility of PROMs to support the development of performance measures.

## Data Availability

The datasets used and/or analysed during the current study are available from the corresponding author on reasonable request.

## Abbreviations

PROs: Patient-reported outcomes
CAT: Computerized adaptive testing
PROMIS®: Patient-Reported Outcomes Measurement Information System®
PROMs: Patient-reported outcome measures
IRT: Item response theory
RIC: Rehabilitation Institute of Chicago
SQL: Structured Query Language
NIH: National Institute of Health
